# Palatability assessment of prescribed diets on domestic shorthair cats

**DOI:** 10.14202/vetworld.2022.640-646

**Published:** 2022-03-23

**Authors:** Nazhan Ilias, Ahmad Harris Hakim Zaki, Awang Hazmi Awang Junaidi, Lau Seng Fong, Ikhwan Saufi, Mokrish Ajat

**Affiliations:** 1 Department of Veterinary Preclinical Sciences, Faculty of Veterinary Medicine, Universiti Putra Malaysia, 43400 UPM, Malaysia; 2 Department of Veterinary Clinical Studies, Faculty of Veterinary Medicine, Universiti Putra Malaysia, 43400 UPM, Malaysia; 3 University Veterinary Hospital, Faculty of Veterinary Medicine, Universiti Putra Malaysia, 43400 UPM, Malaysia

**Keywords:** aroma, domestic shorthair cats, flavor, palatability, prescribed diet

## Abstract

**Background and Aim:**

The value of the pet food industry, which is majorly the prescribed diet, exponentially increased over the years due to increased awareness among pet owners to provide a healthy lifestyle for their pets. However, several factors such as aroma, flavor, texture, and shape of prescribed diets greatly influenced the palatability in cats. Therefore, this study aimed to determine the palatability of the prescribed diet for domestic shorthair (DSH) cats.

**Materials and Methods:**

The two-bowl method was employed to determine the palatability of prescribed diets on five DSH cats for 6 days. Furthermore, the four types of prescribed diet assessed in this study were struvite, renal, hypersensitivity, and intestinal. Furthermore, the pet food palatability was analyzed using “First Approached,” “First Consumed,” “Total Consumption,” and “Intake ratios.”

**Results:**

Our findings revealed that “Total Consumption” and “Intake Ratios” were significantly different in struvite, renal, and intestinal diets compared to the hypersensitivity diet. In addition, this result indicates that the hypersensitivity diet is the most unpalatable compared with other diets.

**Conclusion:**

A detailed investigation of the diet ingredients showed that a hypersensitivity diet lacks herbs and spices than the other diets. Therefore, these ingredients lacking in the hypersensitivity diet influence the palatability of pet foods.

## Introduction

In 2018, the global market for pet care reached U.S.$125 billion, with reported global sales accounting for 73% of the total sales, approximately U.S.$91.1 billion. This figure shows that the pet food industry is gradually expanding and developing globally [1]. Palatability is the perception derived during food consumption accounting for the flavor and the animal’s perception of appearance, temperature, size, texture, consistency, and prior food experiences [2]. Therefore, the prescribed diet is not just a nutrient delivery tool to the animal. Thus, consideration of the animal’s palatability and acceptability during diet production is necessary. A prescribed diet may possess essential nutrients for improving animals’ health; however, its unpalatability may cause the animals’ refusal to consume. Importantly, appearance, aroma, texture, and flavor are sensory characteristics essential in determining pet food acceptability [3]. Therefore, considering these sensory characteristics are vital during the development and production of the prescribed diet. These characteristics increase the diet’s palatability and acceptance [3], facilitating essential nutrient delivery that improves animal health.

Developing a prescribed diet helps improve the animals’ health conditions, such as renal, intestinal, skin, obesity, and hypersensitivity problems. Prescription diets, also known as medical foods, are produced based on the nutritional requirements for diseases. Therefore, these diets can improve the animal’s quality of life and prevent further deterioration of health. Various prescription foods are available in veterinary practices, especially for felines. However, sick cats may be reluctant to consume prescription diets due to issues of unpalatability, consequently resulting in its malnutrition. Therefore, efforts are required to produce prescription diets of high palatability to sick cats, complying with the essential nutrients requirements. The pet food industry is challenged to find an equilibrium between palatability and the nutritional quality of cats’ diets [4].

The smell or odor of the diet’s chemical components influences the olfactory receptors (olfaction) in the nasal cavity. Aroma is a collection of olfactory stimuli from food and the surrounding environment. The vomeronasal or Jacobson’s organ detects aroma in cats and dogs and enhances the olfactory sensing organ [2]. According to Koppel [3], the olfactory sensitivity of cats and dogs is more developed than that of humans, whereas the sensation of taste evolved more in humans. Humans exhibit about 3-4 cm^2^ of olfactory epithelia; whereas cats and dogs exhibit about 21 cm^2^ and 18-50 cm^2^, respectively, including numerous olfaction-related neurons [5]. However, cats possess a strong sense of olfaction that is not advanced compared to dogs that use it for tracking and searching. A cats’ olfactory system is programmed for searching new or untrusted odors. Furthermore, they sniff their food intensely to assess its freshness and quality [2]. Bradshaw proved that response to aroma alone was inadequate in resolving neophobia. For instance, when cats were given a typical commercial food and an artificial lamb flavored food, they chose the former [6].

Therefore, this study determined the prescribed diets with the highest palatability percentage and acceptance among the domestic shorthair (DSH) cats. Determining the palatability and acceptability in these prescribed diets ensures that DSH cats continuously eat, even when sick or diseased, to provide their body with essential nutrients constantly.

## Materials and Methods

### Ethical approval and Informed consent

The use of animals in this study was approved under permissions and the guidelines of the Institutional Animal Care and Use Committee of Universiti Putra Malaysia (UPM) with the Animal Use Protocol (AUP) permission record and reference number UPM/IACUC/AUP-U017/2020. The Institutional Animal Care and Use Committee approved five cats for this study. Verbal consent was obtained from each cat owner before the study.

### Research location, study period, and animals

The study was conducted in the Animal Research Facility, Faculty of Veterinary Medicine, UPM, from 12^th^ August 2020 to 31^st^ December 2020. Five (three males and two females) intact adult (median age of 4 years, 3 months) DSH cats were used. Routine physical examination was performed at the beginning of the study and throughout the experiments. Therefore, they presented with a body condition score of 4-5/9 with a mean bodyweight of 3.9 kg. The cats were placed in an individual cage at the previously mentioned research facility and remained healthy during the study, with no clinical events reported.

### Palatability test

In this study, the two-bowl test was used to test the diet palatability [2]. First, cats were acclimatized to the new environment 3 days before the study. Furthermore, the cats were given two bowls of dry food from different brands and types during the acclimatization period. Food was also given twice daily at 9 am and 5 pm. The type of prescribed diets used in this study includes (A) struvite, (B) renal, (C) hypersensitivity, and (D) intestinal and was obtained from an undisclosed company to protect its commercial rights. First, the struvite diet was used to break up struvite stones, thus reducing their buildup in the body. Next, the renal diet, which contains reduced phosphorus and sodium content, was used to reduce the stress on the kidneys in chronic renal failure cases. Then, a hypersensitivity diet was used in cases of food allergy or intolerances. Finally, the intestinal diet, a highly digestible ingredient for reducing digestive system stress, regulates acute and chronic gastrointestinal disorders. The ingredients used in these four prescribed diets can be referred to in supplementary data (supplementary data can be available from the corresponding author on a reasonable request).

The study was conducted for 6 days, with foods A and B given on the 1^st^ day, foods A and C on the 2^nd^ day, foods A and D on the 3^rd^ day, and foods B and C on the 4^th^ day. Finally, foods B and D were given on the 5^th^ day, and foods C and D were given on the last day. Food was weighed using a digital scale and calculated as recommended by the supplier, sufficient for the cat’s daily requirements.

Two selected food types were randomly placed in respective bowls, presented simultaneously to the animals, and observed for 30 min or until one of the bowls was approached first or consumed first. The bowls were left until the next food was given. During the evening trial, the food was switched sides to avoid establishing a choice bias for the animal. Foods A and B were chosen for day one, followed by foods A and C, foods A and D, foods B and C, foods B and D, and foods C and D, sequentially. The remaining food left before each trial was collected and stored in a vacuum-sealed bag. At the end of trials, the remaining foods were weighed using the same digital scale to determine the most consumed food during the food preparation.

### Human panel sensory assessment

The method from Pickering [7] was adapted for the human assessment. Summarily, the assessment involved the aroma or smell of the food using a human odor panel. Then, the volunteers were required to categorize the food odor according to the scale provided: Number one as the most palatable; fourth as the most unpalatable in terms of human perception of aroma. The survey was conducted with four different pet food, each packed into a sealed bag and labeled as A, B, C, and D. Then, each pet food was smelled by the volunteers and categorized from the most palatable to the most unpalatable. After assessing each sample, volunteers were given a cup of coffee grounds (to smell for one minute) to neutralize the previously smelled pet food odors. In addition, in this study, a human panel was used to test the human sense of smell in assessing the pet food and develop a baseline odor for the tested product.

### Statistical analysis

The “First Approached” and the “First Consumed” were tested using Chi-square, whereas the “Total Consumption” of the food was tested using one-way analysis of variance to compare the four prescribed diets used in this study. The survey on the human subject (human odor assessment) was analyzed using descriptive analysis, and p<0.05 was considered statistically significant. Data were analyzed using the statistical package for the social sciences (SPSS) (SPSS for Windows Ver. 19.0; SPSS Inc., Chicago, IL, USA).

## Results

### First approached

**Figure-1:**
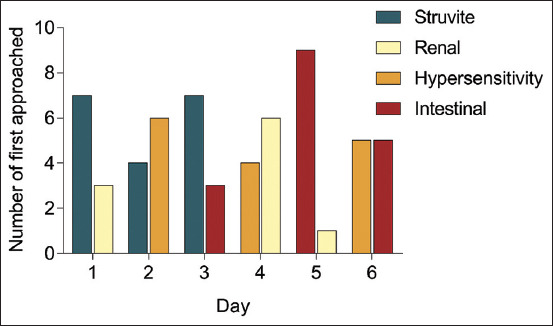
Different prescribed diets “First Approached” by cats according to different days.

The two prescribed diets were compared by observing the diet First Approached or sniffed when both diets were presented to the cats each day. The “First Approached” result is shown in Figure-1. The results showed no superiority in the prescribed diet to the others based on the aroma diet. However, a significant difference was observed in the “First Approach,” which occurred on day 5 between the renal and intestinal diets (p=0.001).

**Figure-2:**
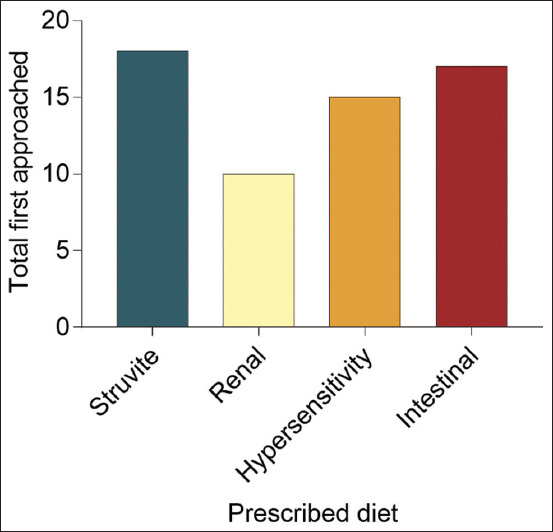
The total number of “First Approached” by domestic shorthair cats according to the types of prescribed diets throughout the study period.

A graph was generated from the “First Approached” results to summarize the number of first approaches according to the type of prescribed feed (Figure-2). Briefly, the renal diet exhibits the least “First Approached” than other diets, whereas the struvite diet exhibits the most “First Approached.” In addition, the data analysis revealed that the aroma of the renal diet was less appealing to the DSH cats than the aroma of other diets.

### Consumed first

**Figure-3:**
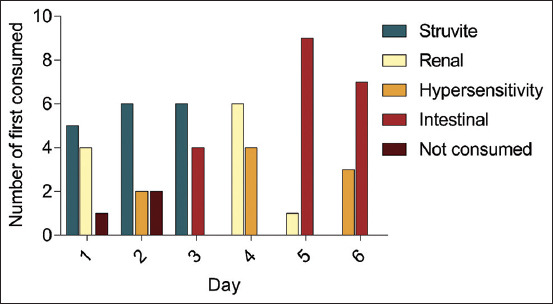
Different prescribed diets “First Consumed” by cats according to different days.

We compared two prescribed diets being “Consumed First” when both diets were presented to the cats daily. The result of the “First Consumed” is shown in Figure-3. According to the results, no prescribed diet was superior to the others in terms of the diet flavor. However, the “Not Consumed” diet was observed when the cats did not consume either one of the diets after 30 min of presentation. The significant differences in the “First Consumed” can be observed between the renal and intestinal diets (p=0.001) on the 5^th^ day of the experiment.

**Figure-4:**
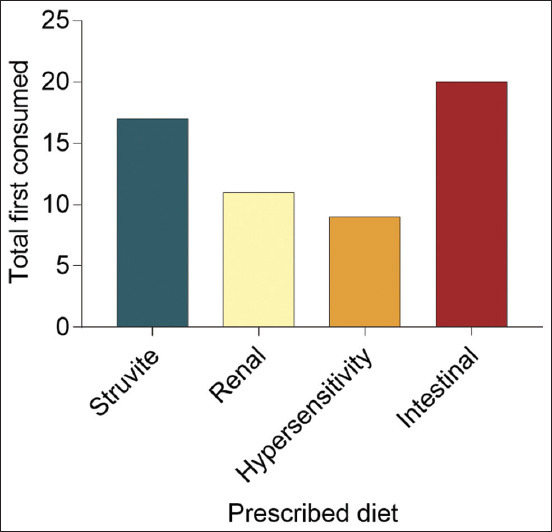
The total number of “First Consumed” according to the types of prescribed diet given to domestic shorthair cats.

The further analysis summarized the “First Consumed” prescribed diets among DSH cats (Figure-4). Furthermore, the hypersensitivity diet demonstrated the lowest “First Consumed” compared to other diets, while the intestinal diet demonstrated the highest “First Consumed.” These observations revealed that the hypersensitivity diet was unpalatable to the DSH cats, whereas the intestinal diet was more palatable when compared with other diets.

### Total consumption

**Figure-5:**
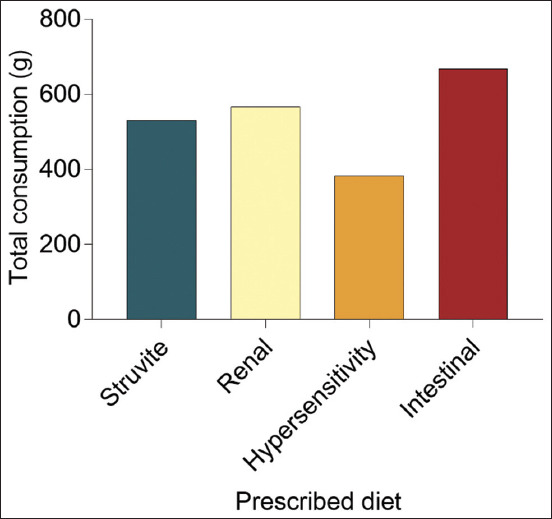
“Total consumption” (g) of each prescribed diet by domestic shorthair cats throughout the study period.

As shown in Figure-5, this observation compared the “Total Consumption” (g) of each prescribed diet used in this study. Results on the “Total Consumption” indicated that the intestinal diet was the highest compared with other diets, whereas the hypersensitivity diet was the lowest. In addition, our observation showed that the intestinal diet was the most palatable, whereas the hypersensitivity diet was most unpalatable compared with the other diets. The difference between the intestinal and hypersensitivity diets was significant statistically (p=0.002).

### Intake ratio

**Figure-6:**
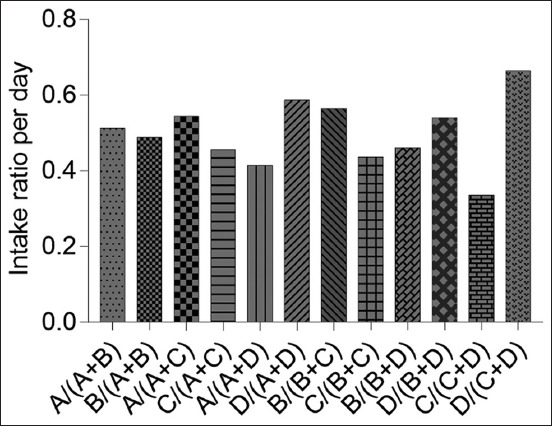
“Intake Ratio” of prescribed diets. A=Struvite, B=Renal, C=Hypersensitivity, and D=Intestinal.

In this study, the “Intake Ratio” (g) described the ratio between the “Total Consumption” of the two prescribed diets presented each day, as shown in Figure-6. According to the “Intake Ratio” results, significant differences were found in the ratio of the hypersensitivity diet to the struvite diet (p=0.002), renal diet (p=0.001), and intestinal diet (p<0.001). These observations showed that the hypersensitivity diet was more unpalatable than the other three diets.

### Survey on humans

A total of 13 correspondents were selected. In this survey, the human subjects were asked to smell the prescribed diets and score them according to the scale, where 1 represents the least pleasant and 4 is the most pleasant. The results of the observation are shown in Table-1.

**Table-1:** Descriptive analysis of prescribed diets’ aroma by human odor assessment.

Type of prescribed diets	Score			

	Least pleasant (1)	Less pleasant (2)	More pleasant (3)	Most pleasant (4)
Struvite	1	3	5	4
Renal	2	4	3	4
Hypersensitivity	9	2	0	2
Intestinal	1	4	5	3
Total correspondent	13	13	13	13

## Discussion

In this study, the assessment of the palatability is based on (1) “First Approached,” (2) “First Consumed,” (3) “Total Consumption,” and (4) “Intake Ratio.” The “First Approached” result indicated that the intestinal diet exhibits a better aroma than the renal diet because cats perceive an advanced olfactory system. Furthermore, cats use the vomeronasal organ, nose, and tongue together to identify the flavor of a compound because of its limited ability for taste utilization due to a low amount of taste buds [8]. This result shows that the food’s aroma is essential in determining which food the cat will approach first. Furthermore, researchers reported that cats use aromas to identify and select food [9]. The “First Approached” result also showed that aroma in the pet food helps determine which food is approached first when given two different types of pet food.

The results from the present study showed that the “First Approached” is consistent with the “First Consumed” data. In addition, the aroma of pet food plays an important role in the choice of food chosen by the cat, which aligns with the previous study results [8]. Therefore, cats use the information from the aroma of food to determine the source of food to be consumed. Therefore, the “First Consumed” result indicated that the intestinal diet differs significantly from the renal diet. More so, based on the cat’s perception, the pet food with the best aroma was approached and consumed first when presented with a choice of food.

In addition, measuring the sensory factors (e.g., taste) of feeding evaluates the combination of the favorable and undesirable experiences resulting from food appearance and consumption [5]. Taste defines the chemoreceptor (taste bud) reaction excitation in the oral cavity [2]. Domestic cats are not drawn to, nor seen to avoid, the taste of sweet carbohydrates and intense sweeteners. However, they are observed to prefer chosen proteins whereas dislike stimuli resulting from either bitter or very sour taste to humans [10]. Substances that taste bitter to humans are commonly avoided in the animal kingdom due to intrinsic plants and animals’ protective mechanisms [11]. The amino acid units in the cat are inhibited by a group of “bitter” amino acids, which include L-tryptophan, L-isoleucine, L-arginine, and L-phenylalanine, which exhibit hydrophobic side chains [6].

Next, the “Total Consumption” and the “Intake Ratio” result determined whether the taste of the pet food is as good as the aroma. The “Total Consumption” indicated that the intestinal diet differed significantly from the hypersensitivity diet. The preference order from the “Total Consumption” result is intestinal, renal, struvite, and hypersensitivity diet. The results also showed that the hypersensitivity diet was the least preferred pet food considering its aroma and flavor than other diets, likely due to the ingredients used in others. In contrast, the hypersensitivity diet lacks herbs and spices compared with other diets.

However, the other three diets contain herbs and spices such as ginger, chamomile, thyme, coriander, rosemary, and *Yucca schidigera*. The herbs and spices can increase pet food palatability by producing a better aroma that attracts the cats to approach, consume, and continuously eat the food. Furthermore, captive felines are reported to demonstrate an arousing impact in the presence of spices such as chili, cinnamon, cumin, nutmeg, and ginger [12]. Similarly, mice and rats demonstrated anxiolytic properties of inhaled valerian, cedarwood, lemon oil, and chamomile [13]. Hence, it can be asserted that herbs and spices positively increase pet food palatability.

Palatability is the experience generated when food is eaten, accounting for the taste and the animal’s perception of appearance, temperature, size, texture, quality, and probably previous experiences [2]. Aroma, flavor, texture, and particle size are additional characteristics contributing to pet food preferential consumption by cats [8]. The palatability of cat foods is improved by moisture, animal fats, protein hydrolysates, meat extracts, and a few amino acids (*e.g*., alanine, histidine, proline, and lysine) [14]. In addition, herbs and spices influence pet food palatability through increased aroma and flavor. The flavor is a mixture of taste sensation and olfactory receptor stimulation in the oral, nasal, and laryngeal cavities [2]. It is crucial because pets consider the first choice to consume based on the odor and continue to choose the food to eat when the taste is as good as the aroma [3].

The “Intake Ratio” is equivalent to the preference ratio, defined as the ratio between the two types of food compared each day. The “Intake Ratio” results were similar to the odor and taste, which play a crucial role in determining food choice to consume. The preference ratio results showed significant differences between the struvite, renal, and intestinal compared with the hypersensitivity diet. In addition, the result demonstrated that the hypersensitivity diet was not the preferred diet but the most unpalatable or the least attractive in terms of the aroma and flavor compared with other diets. According to Hullár *et al*. [9], when a cat considers the aroma of food more tempting than another, it continually consumes it without trying the other food. The most significant flavor enhancers show a “continuous preference effect” in cats when offered a similar diet with the same taste repeatedly, thus representing the final approval of food in the long run [15]. This continuous preference ensures that the cats choose the particular food and continue eating it without exhibiting flavor fatigue for long-term consumption.

The survey was done on humans using their sense of smell to determine if the pet food is acceptable and palatable from the owners’ perceptions. However, the human sense of smell differs from that of cats. It is established that a cat’s sensitivity toward smell is 14 times more accurate than humans [8]. The survey indicated that the hypersensitivity diet was the least favorable and acceptable. Therefore, the pet food must possess an attractive aroma toward the owner since the owner is responsible for buying and determining what type of food needs to be given to their cat. According to Koppel [3], researchers must develop ways to consider the pet’s preference and the owner’s perspective on the pet food into the product concept to ensure continued consumption of the food by the pet.

Furthermore, the owner must be assured that their pet would love the food; otherwise, they should stop purchasing it [2]. These findings show that the owner’s perception of the food is also crucial in determining whether the pet food will be given to the cats or not. Thus, the pet food industry is responsible for producing pet food of high palatability to the cats and acceptable to the pet owner.

The aroma and flavor of pet food are essential factors influencing the “Total Consumption” and the “Intake Ratio” of food. The aroma determines the “First Approach” and the “First Consumed,” whereas the taste determines the cat’s continuity to eating. A single-bowl test (or a single stimulation test), where the subject exhibits unlimited access to a single food for a given period, was primarily used to measure given food acceptability [15]. This test gives an indicator or assumption for “acceptance” but no details on choice, degree of preference, or other hedonic food features [2]. Preference suggests that a decision must be taken between sample foods, which are usually achieved using a two-bowl method [3]. In this approach, two foods are presented separately and concurrently introduced to the animal [2]. In addition, the significant indicators for these assessments involved selecting the first food product to be consumed, thus representing olfactory sensitivity and attractiveness, the quantity of food consumed, the ratio of food consumed, the proportion of food consumption, and the preference ratio [14,15].

## Conclusion

The palatability of pet food, especially when considering the cat’s preference, can be determined using the two-bowl test. This study demonstrated that a few herbs and spices in the diet could significantly increase palatability and consumption of feed among pets. In addition, the human odor panel is greatly limited due to the differences in cat and human odor sensation. However, opportunities also exist to develop a method that uses a human odor panel to create a baseline for pet food’s good and palatable odor.

In contrast, the taste of the pet food is critical for cats to consume or reject the food given continually. The palatability of pet food, especially when considering the cat’s preference, can be determined using the two-bowl test. The “First Approach,” “first consumed,” “Total Consumption,” and “Intake Ratio” provide vital palatability study results. This study demonstrated that a few herbs and spices in the diet could significantly increase palatability, increasing pet food’s “Total Consumption”.

Moreover, opportunities exist to develop a human odor panel to create an acceptable and palatable odor baseline. The present study determined palatability only by the “First Approach,” “First Consumed,” “Total Consumption,” and “Intake Ratio” through two-bowl tests. Assessment of the behavioral response future studies would be beneficial by installing a dashcam inside the cage. Behavior such as nose licking, grooming, biting, lip licking, and moving tail should be observed, as suggested by Bradshaw *et al*. [6]. Furthermore, a larger sample is recommended for more accurate and significant results to support the study. Finally, in place, a 1-day test for each trial, the assessment can last for 2-3 days to measure any side bias and flavor fatigue. Hence, an accurate result can be obtained to determine pet food palatability in the long run.

## Data Availability Statement

Supplementary data can be available from the corresponding author on a reasonable request.

## Authors’ Contributions

MA and LSF: Project administration and resources. MA, LSF, and AHAJ: Conceptualization and supervision. MA, LSF, AHAJ, and IS: Methodology and validation. AHHZ and NI: Investigation. NI, AHHZ, MA, LSF, AHAJ, and IS: Formal analysis and data curation. NI: Software and visualization. AHHZ and NI: Writing-original draft preparation. NI and MA: Writing-review and editing. All authors have read and approved the final manuscript.
